# An LGD model with extrinsic nucleations for polarization dynamics in ferroelectric materials and devices

**DOI:** 10.1038/s41598-025-03469-8

**Published:** 2025-06-04

**Authors:** Mattia Segatto, Daniel Lizzit, David Esseni

**Affiliations:** DPIA, University of Udine, Via Delle Scienze 206, Udine, Italy

**Keywords:** Electrical and electronic engineering, Electronic devices

## Abstract

We present a critical reexamination of the Landau–Ginzburg–Devonshire (LGD) model for ferroelectric materials that is based on intrinsic nucleation events. Theoretical considerations and a systematic comparison with experiments steered us towards a novel version and calibration of the LGD model relying instead on extrinsic nucleations. We show that the new model can not only improve the agreement with experiments, but also help reconcile the interpretation of polarization reversal in poly-crystalline and epitaxial ferroelectrics.

## Introduction

Ferroelectric materials have a wide spectrum of important applications, and have been employed in several energy harvesters, sensors, and actuators, which mainly take advantage of the pyroelectric and piezoelectric properties of ferroelectrics^1^. Coming to applications in the field of the electron devices, lead-titanate oxide Pb(Zr,Ti)O_3_ (PZT) is arguably one of the most studied ferroelectric material due its exploitation in ferroelectric RAMs^1^, where the large remnant polarization of PZT has been used as a robust state variable. However, PZT and other ferroelectric materials belonging to the perovskite family suffer from a difficult integration in CMOS fabrication technologies^1^. Therefore, thanks to the discovery of ferroelectricity in CMOS processing-compatible fluorite-type Hf$_{0.5}$Zr$_{0.5}$O$_2$^2^, and wurtzite-type Al$_{1-x}$Sc$_{x}$N materials^3^, a newfound interest has emerged for ferroelectric materials in electronic devices, such as ferroelectric tunnel junctions, FeFETs, ferroelectric memristors and memcapacitors^4–7^.

Crucial to all applications of ferroelectric materials is the understanding of the Polarization Reversal (PR). These measurements aim to understand the switching dynamics of ferroelectric materials and they are usually carried out by pre-setting the ferroelectric material in a known polarization state, followed by applying an electric field pulse with fixed magnitude and variable duration. The duration of the pulse can extend from hundreds of nanoseconds to tens of milliseconds, depending on the intensity of the applied electric field. After such a pulse, the device is reset by applying a resetting waveform while measuring the switched polarization. With this procedure, it is possible to extract the fraction of switched spontaneous polarization over time for different magnitudes of applied electric fields. In this respect, it has been clearly established by Piezoresponse Force Microscopy measurements that PR in ferroelectrics occurs as a non uniform process^8^, whereby nucleation starts at only a few sites and then expands in larger regions. It is also known that virtually 100% of the nucleation events are extrinsic and occur at lattice defects or at grain boundaries^9^. Moreover, the growth of reversed sites stops at grain boundaries^10^, which is particularly important in poly-crystalline materials. In addition, it has been repeatedly reported in *ab initio* studies that the calculated coercive field, $E_\text {IN}$, for an intrinsic nucleation is much larger than the coercive field, $E_\text {C,exp}$, observed in PR experiments and in measured polarization versus electric field ($P_\text {T}-E_\text {app}$) curves^11,12^. This is again consistent with the extrinsic nature of nucleations.

Despite all the above arguments in favour of the extrinsic nucleation picture, to our best knowledge the Landau–Ginzburg–Devonshire (LGD) equation has been systematically employed assuming an intrinsic nucleation scenario. In this framework, the ferroelectric material parameters are calibrated so as to have an intrinsic coercive field $E_\text {IN}$ equal to the coercive field $E_\text {C, exp}$ observed in PR or $P_\text {T}-E_\text {app}$ experiments^13–16^, whereas extrinsic nucleation events are simply neglected. Furthermore, LGD-based simulations have almost exclusively focused on the interpretation of $P_\text {T}-E_\text {app}$ curves or negative capacitance measurements^13–15,17,18^, with very limited attention devoted to PR experiments^19^.

In this article we first show that an LGD model, when it relies on intrinsic nucleations and it is calibrated against $P_\text {T}-E_\text {app}$ measurements, leads to quite large discrepancies with PR experiments. Then we implement a revised version of the model, whereby the LGD equation describes the propagation of a polarization reversal triggered by extrinsic nucleations. In this novel interpretation and usage of the LGD model, the coercive field for switching propagation, $E_\text {SP}$, can be much smaller than the intrinsic coercive field $E_\text {IN}$. Our results show that the revised model can not only improve the agreement with $P_\text {T}-E_\text {app}$ and PR experiments for three different data sets, but also help elucidate the differences between PR in poly-crystalline and epitaxial ferroelectrics.

## Results and discussion

### Nucleation and propagation in LGD model

The thermodynamic potential employed in the LGD model for a Metal-Ferroelectric-Metal (MFM) structure and assuming ideal metallic electrodes can be written as^13,20^:

1 $$\begin{aligned} U(P,\nabla P)=\int _{V} \left[ \alpha P^2 + \beta P^4 + \gamma P^6 - \dfrac{\varepsilon _0 \varepsilon _{\textrm{FE}}}{2}E_{\textrm{app}}^2 - E_{\textrm{app}} \cdot P + k \, | \nabla P |^2 \right] d{\textbf {r}}dz \end{aligned}$$

where $\textbf{r}=(x,y)$ is the coordinate at the ferroelectric-metal interface and *z* is the abscissa normal to the interface. The spontaneous polarization $P({\textbf {r}},t)$ is here assumed to be aligned with the *z* direction and the dynamic equation reads^21^

2 $$\begin{aligned} \rho \frac{\partial P}{\partial t} = - \frac{\delta U(P,\nabla P)}{\delta P} =-2\, \alpha P - 4 \, \beta P^3 - 6\,\gamma P^5 + E_{\textrm{app}} - 2k \, (\nabla ^2 P) \end{aligned}$$

In Eqs. (1) and (2), $E_\text {app}$ denotes the *z* component of the electric field in the ferroelectric, $\alpha$, $\beta$, $\gamma$ are the anisotropy coefficients of the Landau free energy, $\rho$ is a resistivity setting the time scale $t_{\rho }=\rho /(2|\alpha |)$ of the dynamics, and *k* is the domain wall coupling constant^13,22^. Equation (2) implies two possible mechanisms triggered by a large enough $E_\text {app}$. The first is the Intrinsic Nucleation (**IntNucl**), which is mainly governed by $\alpha$, $\beta$, $\gamma$ and describes the field-driven flipping of an elementary dipole. The second mechanism is the propagation of an extrinsic nucleation, which is mainly governed by the domain wall coupling *k*. In the conventional interpretation and usage of the LGD model, based on the **IntNucl** scenario, the anisotropy coefficients are calibrated so as to reproduce the remnant polarization, $P_\text {R}$, and to have an intrinsic corcive field $E_\text {IN}$ equal to the coercive field $E_\text {C,exp}$ observed in PR and $P_\text {T}-E_\text {app}$ experiements. In the Extrinsic Nucleation (**ExtNucl**) scenario proposed in this work, instead, the LGD model is used to describe the propagation of the polarization reversal triggered by extrinsic nucleations. The calibration of the anisotropy coefficients and of the domain wall coupling *k* will be discussed in **Extrinsic Nucleation LGD Model** subsection.

### Simulations and comparison to experiments

In this section we benchmark the **IntNucl** and **ExtNucl** version of the LGD model by comparing simulations with experiments. In particular, we selected data sets reporting both $P_\text {T}-E_\text {app}$ and PR data for the same samples, and we investigate whether it is possible to find a good agreement with both the $P_\text {T}-E_\text {app}$ and PR data using the same set of ferroelectric parameters.

In this work, we numerically solved Eqs. (1) and (2) by using a real-space discretization scheme, leading to

3 $$\begin{aligned} U =&\sum _{\textrm{i}=1}^{n_S}\left[ \left( \alpha _\textrm{i} P_\textrm{i}^2 +\beta _i P_\textrm{i}^4 +\gamma _\textrm{i} P_\textrm{i}^6\right) -\dfrac{\varepsilon _0\varepsilon _{\textrm{FE}}}{2}E_{\textrm{app}}^2-E_{\textrm{app}}P_\textrm{i}+\dfrac{k}{2d^2}\sum _{\textrm{ni}}\left( P_{\textrm{i}}-P_{\textrm{ni}}\right) ^2\right] \end{aligned}$$

4 $$\begin{aligned} \rho \frac{\partial P_\textrm{i}}{\partial t} =&-2\alpha _\textrm{i}P_\textrm{i}-4\beta _\textrm{i}P_\textrm{i}-6\gamma _\textrm{i}P_\textrm{i} + E_{\textrm{app}} - \dfrac{2k}{d^2}\sum _{\textrm{ni}}(P_{\textrm{i}}-P_{\textrm{ni}}) \end{aligned}$$

where *d* is the size of the elementary polarization site, $\alpha _i$, $\beta _i$, $\gamma _i$ are the anisotropy coefficients that can vary from site to site, and $n_S$ is the total number of sites. To reproduce epitaxial ferroelectric materials we impose periodic boundary conditions to simulate large effective areas with lower computational effort, while for poly-crystalline ferroelectric materials we impose that the propagation stops at the border of the simulated area. Further details regarding the simulation framework can be found in the Supplementary Information. Experimental data show that the electric field controls the switching time, following a nucleation–limited switching scenario. As a result, the voltage dependence of the viscosity parameter is necessary for the switching dynamics of widely used ferroelectric materials. In all simulations we used an $E_\text {app}$-dependent resistivity $\rho$ according to the Merz’ law^23,24^

5 $$\begin{aligned} \rho = \rho _0\,\text {exp}\left( \frac{E_{\textrm{a}}}{E_{\textrm{app}}}\right) \end{aligned}$$

and the parameters for the activation fields $E_\text {a}$ can be found in Table 1. The different values of the anisotropy coefficients $\alpha$, $\beta$ and $\gamma$ in Table 1 when the same ferroelectric material is described by either the intrinsic or the extrinsic nucleation model are due to the corresponding different values of $E_\text {IN}$ employed in the two scenarios, as we will further clarify in next sections.

**Table 1:** Parameters for the LGD model extracted from a comparison between our simulations and several sets of experimental data. Parameters for the IntNucl LGD scenario correspond to comparisons in Fig. 2, whereas parameters for the ExtNucl LGD refer to Fig. 4. $E_\text {IN}$ is the mean value of the coercive field for intrinsic nucleation and $\sigma {_E}_{\text {IN}}$ is the corresponding standard deviation (normalized to $E_\text {IN}$) for a site-to-site Gaussian distribution of the $E_\text {IN}$ values. $P_\text {R}$ and *k* are defined in the text, while $\rho _0$ and $E_\text {a}$ describe the $\rho$ dependence on $E_\text {app}$ according to Merz’ law in Eq. (5).

	IntNucl: LGD parameters. Data from^25^ for poly-crystalline $\hbox {Si:HfO}_2$ Simulations in Figs. 2a and 2b.	IntNucl: LGD parameters. Data from^26^ for epitaxial $\hbox {Pb(Zr}_{0.4}\hbox {Ti}_{0.6})\hbox {O}_3$ Simulations in Figs. 2c and 2d.	ExtNucl: LGD parameters. Data from^26^ for epitaxial $\hbox {Pb(Zr}_{0.4}\hbox {Ti}_{0.6})\hbox {O}_3$ Simulations in Figs. 4a and 4b.	ExtNucl: LGD parameters. Data from^25^ poly-crystalline $\hbox {Si:HfO}_2$ Simulations in Figs. 4c and 4d.
$E_{IN}$ [MV/cm]	1.12	0.15	0.6	4
$\sigma _{EIN}$ [%]	30	30	1	1
$P_r$ [$\mu$C/cm$^2$]	16	70	70	16
$E_\text {a}$ [MV/cm]	1.9	0.9 @ $E_{app}<0.25$ MV/cm 2.8 @ $E_{app} \ge 0.25$ MV/cm	0.6 @ $E_{app}<0.2$ MV/cm 2.65 @ $E_{app} \ge 0.25$ MV/cm	1.9
$\rho _0$ [$\Omega$m]	1500	$0.012 @ E_{app} < 0.25$ MV/cm $12 @ E_{app} \ge 0.25$ MV/cm	$0.01 @ E_{app}<0.25$ MV/cm $5 @ E_{app} \ge 0.25$ MV/cm	10
*k* [m$^3$/F]	0	$4.55\cdot 10^{-11}$	$4.55\cdot 10^{-11}$	$1\cdot 10^{-9}$
$\alpha$ [m/F], $\beta$ [m$^5$/(FC$^2$)], $\gamma$ [m$^9$/(FC$^4$)]	$-7.89\cdot 10^8$, $8.94\cdot 10^9$, $1.69\cdot 10^{11}$	$-8.45\cdot 10^3$, $-5.88\cdot 10^7$, $7.99\cdot 10^7$	$-3.38\cdot 10^4$, $-2.35\cdot 10^8$, $3.19\cdot 10^8$	$-2.47\cdot 10^9$, $2.79\cdot 10^{10}$, $5.27\cdot 10^{11}$

### Intrinsic nucleation LGD model

**Fig. 1:**
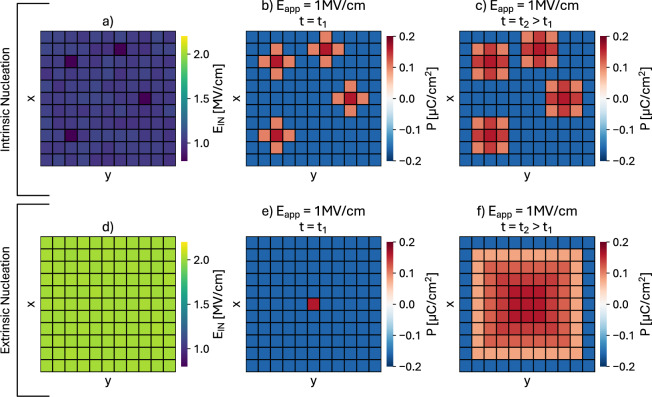
(**a**–**c**) Sketch illustrating the simulation conditions and the ferroelectric dynamics for an **IntNucl**. (**a**) Site-to-site variations of $E_\text {IN}$ are significant compared to the mean $E_\text {IN}$ value; (**b**) upon the application of an electric field ramp, intrinsic nucleations at time $t_1$ occur at the sites having the smallest $E_\text {IN}$; (**c**) propagation at time $t_2>t_1$ of the intrinsic nucleations occurred at time $t_1$. (d)-(f) Sketch illustrating the **ExtNucl** scenario. (**d**) Site to site variations of $E_\text {IN}$ are negligible compared to the mean $E_\text {IN}$ value; (**e**) an extrinsic nucleation occurs at time $t_1$; (**f**) propagation at time $t_2>t_1$ of the extrinsic nucleation occurred at time $t_1$.

**Fig. 2:**
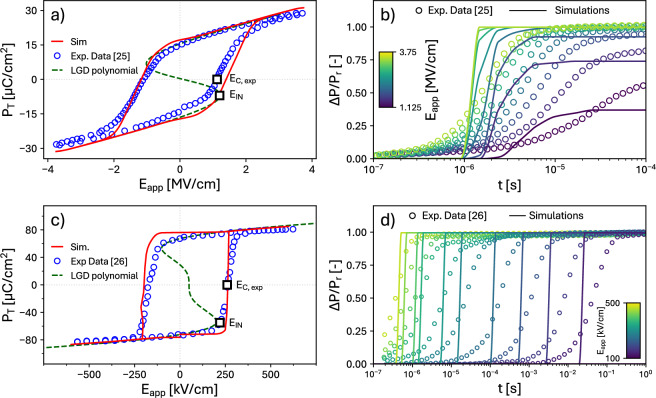
Simulations based on the **IntNucl** LGD scenario. Comparison between simulations and experiments for: (**a**,**b**) poly-crystalline, Si-doped HfO$_2$ capacitors^25^, representative of an NLS-like behavior; (**c**,**d**) epitaxial Pb(Zr$_{0.4}$Ti$_{0.6}$)O$_3$ thin films^26^, representative of a KAI-like behavior. (**a**) Total polarization $P_\text {T}$ versus applied field $E_\text {app}$ for a $P_\text {T}-E_\text {app}$ simulation setup. The dashed line shows the plot of the $E_\text {app}$ versus $P_\text {T}$ quasi-static relation corresponding to the 6-th order polynomial of the LGD model, showing an $E_\text {IN}$ very close to $E_\text {C,exp}$. $E_\text {IN}$ is the mean value of the coercive field in the calibrated LGD model, while $E_\text {C,exp}$ is the coercive field observed in experiments. (**b**) Corresponding polarization reversal simulations for a field $E_\text {app}$ ranging from 1.125 to 3.75 MV/cm. (**c**,**d**) report a similar analysis as respectively in (**a**) and (**b**), but for the experimental data in^26^. (**c**) Total polarization $P_\text {T}$ versus applied field $E_\text {app}$ for a $P_\text {T}-E_\text {app}$ simulation setup. (**d**) Corresponding polarization reversal simulations for a field $E_\text {app}$ ranging from 100 to 500 kV/cm. All simulation parameters are reported in Table 1.

In the **IntNucl** scenario, the mean values of the anisotropy coefficients of the LGD model are calibrated so as to have $E_\text {IN}$ equal to the $E_\text {C,exp}$ observed in experiments. Then site-to-site variations of $E_\text {IN}$ are introduced so that, for a given applied field $E_\text {app}$, intrinsic nucleations start at the sites having the smallest $E_\text {IN}$ and then can propagate to adjacent sites, depending on the value of the domain wall coupling factor *k*. This scenario is depicted in Fig. 1a–c.

Figure 2a shows a comparison between **IntNucl** LGD simulations and experiments for the poly-crystalline, Si-Doped HfO$_2$ samples in^25^, where the total polarization is defined as $P_{\textrm{T}}=P+ \varepsilon _{0} (\varepsilon _{\textrm{FE}}-1)$ $E_\text {app}$, with $\varepsilon _{\textrm{FE}}$ being the background polarization constant. The corresponding values of $E_\text {IN}$ and $P_\text {R}$ are reported in Table 1. In the LGD model, the relation between $P_\text {T}$ and $E_\text {app}$ is obtained by setting $dP/dt$ $=$0 in Eq. (2) and for a homogeneous polarization, which readily gives

6a $$\begin{aligned} E_\text {app} = 2\alpha P+4\beta P^3+6\gamma P^5 \end{aligned}$$

6b $$\begin{aligned} P_\text {T} = P + \varepsilon _0\varepsilon _{\text {FE}} E_\text {app} \end{aligned}$$

Consistently with^25,27^, in Figs. 2a and 2b we assumed a negligible domain wall propagation and set $k=0$. Table 1 and Fig. 2a shows that the mean $E_\text {IN}$ value extracted from the model calibration is close to $E_\text {C,exp}$ and, in particular, it is much smaller than the $E_\text {IN}$ values predicted by *ab initio* calculations, which exceed 5 MV/cm^11,12^. Figure 2b shows that, for the same set of ferroelectric parameters, simulations exhibit quite large discrepancies with the PR experiments carried out on the same sample as in Fig. 2a^25^. This is because, in the **IntNucl** LGD model, the PR simulations are governed by a single time constant (for a fixed $E_\text {app}$), whereas the extrinsic nucleation times in poly-crystalline materials are known to vary over a broad range^10^.

Moving now to an epitaxial material, Figs. 2c and 2d report a similar analysis for the epitaxial PZT samples in^26^. Even in this case, the extracted $E_\text {IN}$ for the **IntNucl** model is close to $E_\text {C,exp}$ and much smaller than predicted by *ab-initio* calculations^28^. Here we used the *k* value estimated in^29^, which results in a quite steep transition in $P_\text {T}-E_\text {app}$ curves, despite the site to site dispersion of the anisotropy coefficients. The agreement with PR experiments in Fig. 2d is again poor, particularly at small $E_\text {app}$. In this case, this is mainly due to the influence of *k* on the polarization dynamics, which results in much steeper transitions compared to experimental PR curves.

### Extrinsic nucleation LGD model

**Fig. 3:**
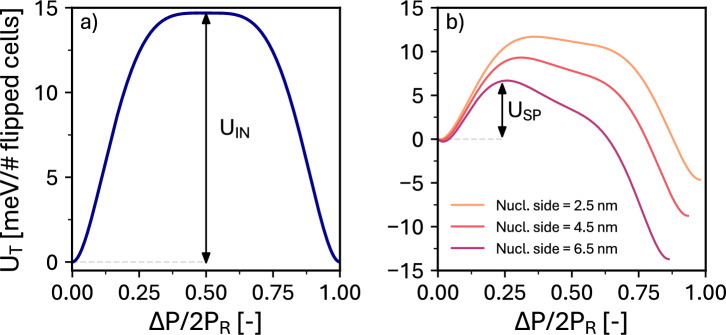
Energy landscapes at $E_\text {app}$ $=0$ for different polarization transitions. (**a**) Reports the energy barrier $U_\text {IN}$ which would be required to obtain an intrinsic nucleation; (**b**) Reports the corresponding energy barrier $U_\text {SP}$ which is needed to overcome in order to have the propagation of the nucleation. Depending on the size of the nucleated side, $U_\text {SP}$ can be much lower than $U_\text {IN}$.

**Fig. 4:**
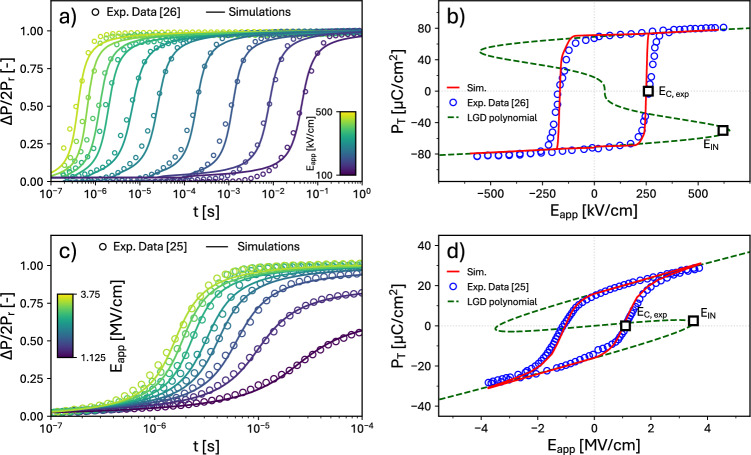
Simulations based on the **ExtNucl** LGD scenario. Comparison between simulations and experiments for: (**a**,**b**) epitaxial Pb(Zr$_{0.4}$Ti$_{0.6}$)O$_3$ thin films^26^, representative of a KAI-like behavior; (**c**,**d**) Si-doped HfO$_2$ ferroelectric material representative of an NLS-like behavior^25^. (**a**) Polarization reversal analysis with $E_\text {app}$ ranging from 100 to 500 kV/cm. (**b**) Total polarization $P_\text {T}$ versus $E_\text {app}$, calculated from Eqs. (6a) and (6b). The dashed line shows the plot of the $E_\text {app}$ versus $P_\text {T}$ quasi-static relation corresponding to the 6-th order polynomial of the LGD model, showing an $E_\text {IN}$ much larger than $E_\text {C,exp}$; (**c**) Polarization reversal measurements and simulations for poly-crystalline, Si-doped HfO$_2$ capacitors from^25^ with $E_\text {app}$ ranging from 1.125 to 3.75 MV/cm. (**d**) Corresponding total polarization $P_\text {T}$ versus $E_\text {app}$ characteristic for the Si-doped HfO$_2$, calculated from Eqs. (6a) and (6b). The dashed line reports the $E_\text {app}$ versus $P_\text {T}$ quasi-static relation, showing an $E_\text {IN}$ much larger than $E_\text {C,exp}$. In all **ExtNucl** simulations we used an extrinsic nucleation density of $3.26\cdot 10^{11}$ cm$^{-2}$ and a nucleated area of 6.25 nm$^2$, which corresponds to a nucleated side of 2.5 nm.

An illustration of nucleations and propagation in the **ExtNucl** scenario is shown in Fig. 1d to f. In this work, we employed a Lorentzian probability density for extrinsic nucleation times^30^

7 $$\begin{aligned} L(\log _{10}(t_{\textrm{N}})) = \dfrac{A\, w}{\pi \,\left[ \left( \log _{10}(t_{\textrm{N}})-\log _{10}(t_{\textrm{M}})\right) ^2+w^2 \right] } \end{aligned}$$

where $t_\text {M}$ is the mean time of the nucleations for a given electric field, *w* is the half-width at half-maximum of the Lorentzian and *A* is a normalization constant.

In our simulation procedure, the time evolution described by the LGD model is periodically stopped, and then new extrinsic nucleation sites are generated according to the Lorentzian distribution of nucleation times $t_{\text {N}}$ given by Eq. (7). The next nucleation site is chosen as the center of a domain in the simulated area that has not yet experienced a nucleation event. Such new sites are then used as initial conditions when the time evolution is restarted and the system evolves according to the dynamics described by the LGD formalism (Eq. (4)).

Figure 3 shows some zero field energy landscapes calculated by varying the site polarization in Eq. (3), and corresponding to either the intrinsic nucleation of a new site (Fig. 3a, energy barrier $U_\text {IN}$), or the propagation of a nucleated site to adjacent sites (Fig. 3b, energy barrier $U_\text {SP}$). The barrier $U_\text {SP}$ for the propagation is shown to be substantially lower than $U_\text {IN}$, so that the corresponding coercive field $E_\text {SP}$ is smaller than $E_\text {IN}$.

Hence, in the **ExtNucl** LGD model we calibrated the anisotropy coefficients to reproduce $P_\text {R}$ and, at the same time, to have a propagation coercive field $E_\text {SP}$ close to the experimental coercive field $E_\text {C,exp}$. This implies that the corresponding $E_\text {IN}$ of the model is instead much larger than $E_\text {C,exp}$, which is in qualitative agreement with recent findings for HfO$_2$^12^.

The density of the extrinsic nucleation sites per unit area is an input parameter for the **ExtNucl**, as it provides the total number of nucleated sites in a given simulated area. In all simulations of Fig. 4 we employed a density of $3.26\cdot 10^{11}$ cm$^{-2}$ and a nucleated area of 6.25 nm$^2$, which are consistent with values reported in literature^8,31,32^. The area of the extrinsic nucleation site employed in Fig. 4 is 6.25 nm$^2$. The influence of the nucleated area on the simulation results will be further discussed in Fig. 6.

**Fig. 5:**
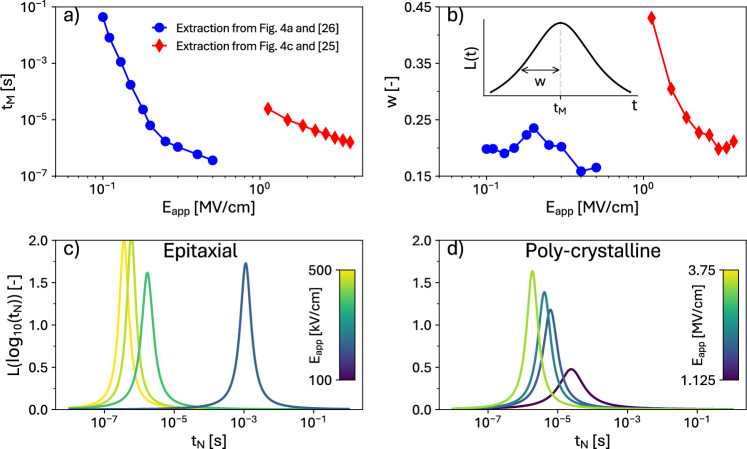
Parameters of Lorentzian function in Eq. (7) describing the distribution of the nucleation times $t_\text {M}$. The parameters are extracted from the calibration of the **ExtNucl** LGD model to experiments. (**a**) $t_\text {M}$ versus $E_\text {app}$. (**b**) The half width at half-maximum *w* of the Lorentzian distribution versus $E_\text {app}$. Corresponding Lorentzian distributions for nucleation times at different $E_\text {app}$ for: (**c**) epitaxial PZT thin-films^26^, the corresponding simulations are shown in Fig. 4a); (**d**) poly-crystalline Si-doped HfO$_2$^25^, the corresponding simulations are shown in Fig. 4c.

Figure 4a shows that the agreement of the **ExtNucl** LGD simulations with the PR experiments in^26^ is better compared to the results of the **IntNucl** model in Fig. 2d. All simulation parameters are reported in Table 1. Moreover, the parameters of the Lorentzian function for the extrinsic nucleation times are also displayed in Fig. 5. Figure 4b shows that the LGD parameters used for PR experiments can reproduce well also the $P_\text {T}-E_\text {app}$ curves. As it can be seen, $E_\text {IN}$ is larger than the $E_\text {app}$ values experimentally spanned in^26^, whereas the corresponding $E_\text {SP}$ is approximately 150 kV/cm. This picture is consistent with the **ExtNucl** scenario, whereby the electric field can propagate extrinsic nucleations, but intrinsic nucleations are instead negligible compared to the extrinsic ones.

Figure 4c shows that, even for the PR dataset in^25^, the **ExtNucl** LGD model can reproduce experiments better than the **IntNucl** counterpart in Fig. 2b. Table 1 shows that, for the **ExtNucl** results, a non-null *k* value between sites is necessary to propagate extrinsic nucleations. This is consistent with^12^, where it has been demonstrated that the polarization switching based on nucleation and growth is indeed possible in HZO and doped HfO$_2$.

Figure 4d reports a good agreement also between simulations and experimental data of $P_\text {T}$ versus $E_\text {app}$ curves for poly-crystalline Si-doped HfO$_2$ in the context of the **ExtNucl** scenario. The simulated results shown in this figure are obtained with the procedure explained in the Supplementary Information.

Figure 5 displays the field dependent parameters $t_\text {M}$ and *w* of the Lorentzian functions entering Eq. (7). For the data in^26^, Fig. 5b reveals that the *w* is fairly independent of $E_\text {app}$, and Fig. 5c shows that the corresponding nucleation times, $t_\text {M}$, have a small dispersion around the mean value $t_\text {M}$. This behavior is representative of a KAI scenario dominated by a single time constant^33^, that in our model is the propagation time scale governed by the LGD equation. Figure 5b also shows that for the data in^25,34^, instead, the *w* steeply increases at small $E_\text {app}$, corresponding to a large dispersion of nuclation times in Fig. 5d. This is representative of the NLS scenario for PR^10^, whereby the PR curves are shaped by a broad dispersion of nucleation times. Similar results were found for the data in^34^, as it is reported in the Supplementary Information along with a comparison between simulations and experimental data of polarization reversal measurements to further validate our model. Our analysis suggests that even epitaxial materials can exhibit an NLS behavior in PR experiments at small $E_\text {app}$, which is consistent with the conclusions in^34^. Moreover, we have shown that an LGD equation based on the **ExtNucl** formulation can describe equally well both the KAI and the NLS scenarios, upon appropriate calibration of the model.

**Fig. 6:**
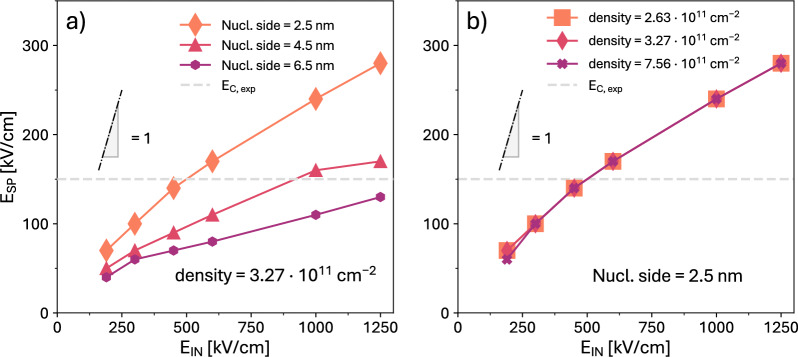
Electric field needed to obtain the full propagation of an extrinsic nucleation ($E_\text {SP}$) versus different intrinsic nucleation electric fields ($E_\text {IN}$). The analysis is performed for different nucleated areas (**a**) and progressively larger nucleation densities (**b**). For a given $E_\text {IN}$ and a given density, increasing the nucleated area results in a lower $E_\text {SP}$. The nucleated side refers to the length of the side of the extrinsic nucleation. In simulations, all extrinsic nucleations are square. The influence of the nucleation density in the range examined is negligible, where the nucleation density is defined as one nucleation (regardless of the nucleation side) over the simulated area.

Figure 6 reports an analysis performed within the context of the **ExtNucl** scenario with periodic boundary conditions, where we investigated the influence of different simulation parameters on the switching propagation field $E_\text {SP}$. In particular, we report simulated $E_\text {SP}$ versus $E_\text {IN}$ curves obtained for different nucleation densities and different nucleated areas.

For a given $E_\text {IN}$ and nucleation density, the increase of the nucleation area leads to a decrease of $E_\text {SP}$ . This can be explained with the support of Fig. 3b, where we observe that a larger nucleated area (corresponding to more elementary sites) results in a lower energy barrier $U_\text {SP}$ for the switching propagation. Such a smaller $U_\text {SP}$, in turn, translates into a lower $E_\text {app}$ to obtain the full switching of the simulated area. The effect of the nucleation density is instead almost negligible in the explored range of densities. This is because the densities analyzed in Fig. 6b, roughly corresponding to the values estimated in^8,31,32^, are low enough that there is no significant interaction or collaboration in the switching propagation of different nucleated sites. Therefore, the $E_\text {app}$ required for a complete switching of the ferroelectric layer is not appreciably reduced by increasing the nucleation density.

## Conclusions

Based on a systematic comparison of numerical simulation results to $P_\text {T}-E_\text {app}$ and polarization reversal experiments for either poly-crystalline or epitaxial ferroelectrics, we have proposed, implemented and calibrated a new version of the LGD model based on extrinsic nucleations. The model shows an improved agreement with experiments compared to the conventional LGD model relying on intrinsic nucleations. The new use of the LGD equations developed in this paper can be applied to any ferroelectric material. This versatility of the model has been demonstrated by showing the comparison between experimental data and simulations for three different ferroelectric materials, namely Pb(Zr$_{0.4}$Ti$_{0.6}$)O$_3$ from^26^ and Si-doped HfO$_2$ from^25^, as well as epitaxial BFO from^34^ in the Supplementary Information.

Our model comes with some limitations as well. First, because our reinterpretation of the LGD theory for ferroelectricity employs the dynamic equations to describe the propagation of extrinsic nucleations, the model is best suited to deal with ferroelectric materials where the polarization dynamics is limited by propagation. This is the case, for example, for the epitaxial Pb(Zr$_{0.4}$Ti$_{0.6}$)O$_3$ layer analyzed in the previous section. In poly-crystalline ferroelectrics, the dynamics is frequently limited by the rate of extrinsic nucleations, as in the case of the Si-doped HfO$_2$ analyzed in the main text. Indeed, our reinterpretation of the LGD model describes the field-driven propagation of an extrinsic nucleation inside the ferroelectric material. When the propagation of a nucleation becomes negligible due to the poly-crystalline nature of the ferroelectric material, a simpler NLS model may be used to obtain qualitatively similar results^10,25^. A second noteworthy aspect is that our model cannot be immediately applied for an arbitrary time–dependent applied voltage, as also discussed in the Supplementary Information.

Overall, we are persuaded that an application of the LGD equations relying on extrinsic nucleations can help us put the modeling of ferroelectric materials on a sounder physical basis, which is also a pre-requisite for a more effective approach to the design of ferroelectric devices.

## Supplementary Information

Supplementary Information.

## Data Availability

All data generated or analysed during this study are included in this published article (and its Information files).
