# Supplementary material for: An LGD model with extrinsic nucleations for polarization dynamics in ferroelectric materials and devices
Source: Sci Rep. 2025 Jun 4;15:19619. doi: 10.1038/s41598-025-03469-8 (PMC12137572; doi:10.1038/s41598-025-03469-8)
Supplement: Supplementary file 1 — Supplementary Information. [file 41598_2025_3469_MOESM1_ESM.pdf]

An LGD model with Extrinsic Nucleations for Polarization Dynamics in Ferroelectric Materials and Devices (Supplementary Information)

Mattia Segatto,* Daniel Lizzit, and David Esseni

1 Polarization reversal and phenomenological models

Polarization reversal is a crucial measurement, which is employed to characterize the dynamics of the ferroelectric materials over a broad range of switching times. The polarization dynamics extracted from polarization reversal measurements in either epitaxial ferroelectrics or poly-crystalline ferroelectrics are typically described with two different phenomenological models.[1–4] In epitaxial materials, polarization reversal is described in terms of the Kolmogorov-Avrami-Ishibashi (KAI) model[1]

$$P(t) = 2P_r \cdot \left\{ 1 - \exp\left[\left(-\frac{t}{\tau} \right)^n \right] \right\} \tag{1}$$

where n is the dimension of the domain growth (usually $n = 2$) and τ is the characteristic switching time, that is known to depend on the nucleation rate and the domain wall velocity[3]. The value of τ is typically estimated by comparing to measurements and, more precisely, it is extracted as the time when the polarization reversal reaches the 63% threshold. The dependence of τ on the applied electric field E_{app} can be expressed with the Merz' Law[5]

$$\tau = t_0 \cdot \exp\left(\frac{E_{\mathrm{a}}}{E_{app}} \right) \tag{2}$$

where t_0 is a fitting pre-factor[3], and E_{a} is the so-called activation field, which can be extracted as the slope of the experimental $\log_{10}(\tau)$ versus $1/E_{\mathrm{app}}$ characteristic. The idea behind the KAI model is an unrestricted propagation of a nucleation event in infinite size crystal[1], and it is usually applied for single-crystal or epitaxial materials having very large grain sizes[6]. This model, however, proves to be ineffective in the description of poly-crystalline ferroelectric materials, especially at very low applied electric fields. In more general terms, the model is not suitable to describe experimental datasets where the polarization reversal appears to occur with a broad range of time constants[2].

To overcome the limitations of the KAI model in poly-crystalline ferroelectric materials, the Nucleation-Limited Switching (NLS) model was proposed[2,7]. In the NLS physical picture, the KAI description of polarization reversal is still valid inside a single grain (or domain). However, differently from the KAI model, the extrinsic nucleations inside a single grain cannot lead to the switching of a large portion of the ferroelectric material (or even the entire material), because a large number of grain boundaries tend to stop the propagation of the nucleation. Under these circumstances, the dynamics of the polarization reversal is not rate limited by the propagation of the nucleation, but by the rate of nucleation events. In fact, in consideration of the relatively small grain size, the propagation of a nucleation inside a single grain is considered as almost instantaneous in the NLS framework. The equation for polarization reversal in the NLS model reads

$$P(t) = 2P_r \cdot \int_{-\infty}^{+\infty} \left\{ 1 - \exp\left[\left(-\frac{t}{t_N} \right) \right] \right\} F(\log_{10}(t_N))\, d(\log_{10}(t_N)) \tag{3}$$

with t_N being the nucleation time and $F(-)$ the probability density function for the nucleation times. Such a probability density is usually assumed to have a Lorentzian form[7,8]

$$L(\log_{10}(t_N)) = \frac{A\,w}{\pi \left[(\log_{10}(t_N) - \log_{10}(t_M))^2 + w^2 \right]} \tag{4}$$

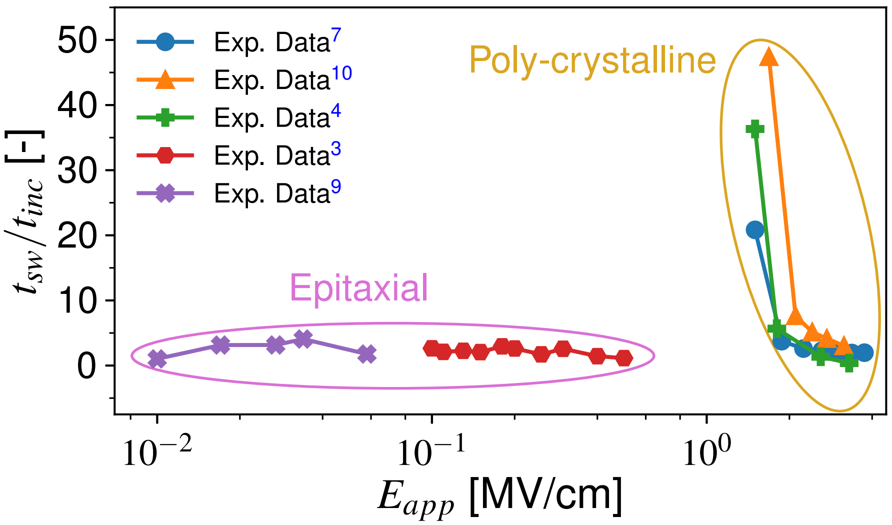

Figure S1. Ratio between incubation time, t_{inc}, and switching time t_{sw} defined in the text versus the applied field, E_{app}, and for polarization reversal experiments. Data from[3,9] correspond to epitaxial ferroelectric materials displaying a KAI-like behaviour, whereby the $t_{\mathrm{sw}}/t_{\mathrm{inc}}$ ratio is fairly independent of E_{app}. Data from[4,7,10] correspond to poly-crystalline ferroelectric materials displying a NLS-like behaviour. For poly-crystalline materials we observe a larger dependence of the $t_{\mathrm{sw}}/t_{\mathrm{inc}}$ ratio on E_{app}, particularly at small E_{app} values.

where t_{M} is the mean time of the nucleations, w is defined as the half-width-at-half-maximum of the Lorentizan and A is a normalization constant.

Figure S1 reports an analysis of the switching times showing a clear difference between the KAI and NLS behaviour in polarization reversal measurements. In this figure, we have introduced an incubation time, t_{inc}, defined as the time needed for the polarization reversal to reach the 20% of the overall switched polarization, and then a switching time, t_{sw}, defined as the time required to go from the 20% to the 80% of the overall polarization reversal. Figure S1 reveals that the KAI-like behaviour is identified by a $t_{\mathrm{sw}}/t_{\mathrm{inc}}$ ratio that is fairly independent of the applied field E_{app} (data from[3,9] corresponding to epitaxial materials), meaning that the polarization versus time curve is quite rigidly shifted when E_{app} is varied. In the NLS-like behaviour instead (data from[4,7,10] for poly-crystalline ferroelectric materials), the $t_{\mathrm{sw}}/t_{\mathrm{inc}}$ ratios are similar to the KAI-like behaviour for the largest E_{app}, but the $t_{\mathrm{sw}}/t_{\mathrm{inc}}$ ratio steeply increases to much larger values at relatively low fields.

2 Simulation Framework and Boundary Conditions

In our simulation framework based on the Landau-Ginzburg-Devonshire (LGD) theory, the ferroelectric dynamics is governed by the equation[11–13]

$$\rho \frac{dP}{dt} = -\frac{\delta G}{\delta P} = -\left[\frac{\partial g}{\partial P} - \nabla \cdot \left(\frac{\partial g}{\partial \nabla P}\right)\right] \tag{5}$$

where we assume that the spontaneous polarization, P, and the applied electric field, E_{app}, are aligned and parallel to the z-axis. The Gibbs' energy functional $g(P, \nabla P)$ employed in this work is given by[11,12,14]

$$g(P, \nabla P) = \alpha P^2 + \beta P^4 + \gamma P^6 + k|\nabla P|^2 - \frac{1}{2}\varepsilon_0 \varepsilon_F E_{\mathrm{app}} - P E_{\mathrm{app}} \qquad [\mathrm{J/m^3}] \tag{6}$$

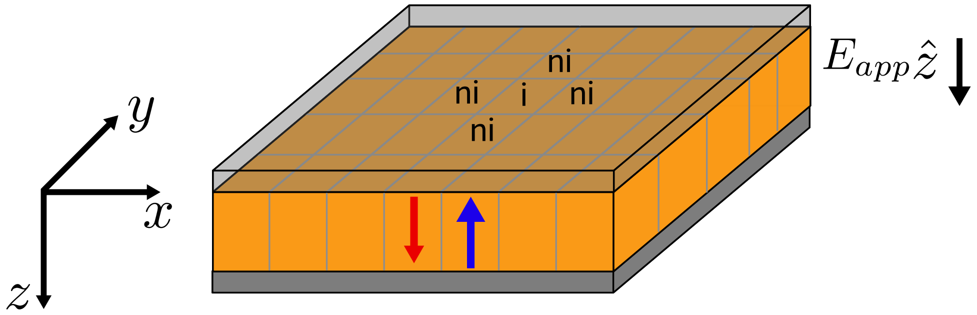

Figure S2. Sketch of a ferroelectric capacitor. The polarization is considered positive (red arrow) when aligned with the versor z, otherwise it is considered negative. Elementary sites obtained by discretization are labeled with the letter i. The neighboring sites used in the discretized version of the Laplacian operator are labeled with the subscript *ni* and are used in Eq. (8) and in Equations (3) and (4) of the main text.

where $\delta G/\delta P$ denotes the variational derivative of $G{=}\int_{\mathrm{Vol}} g\, d\mathbf{V}$ with respect to the spontaneous polarization P. For the Gibbs' free energy functional in Eq. (6), Eq. (5) becomes

$$\rho \frac{dP}{dt} = -2\alpha P - 4\beta P^3 - 6\gamma P^5 - 2k\nabla^2 P + E_{\mathrm{app}} \tag{7}$$

Eq. (7) is then solved numerically by using a spatial discretization scheme in the (x,y) plane of the ferroelectric interface, thus leading to

$$\rho \frac{dP_i}{dt} = -2\alpha_i P_i - 4\beta_i P_i^3 - 6\gamma_i P_i^5 - 2\frac{k}{d^2}\sum_{\mathrm{ni}} (P_{\mathrm{ni}} - P_i) + E_{\mathrm{app}} \tag{8}$$

where d is the side of the discretization site. It can be readily seen that, by employing a forward derivative scheme along both x and y, the discretized Laplacian operator becomes proportional to $\sum_{\mathrm{ni}} (P_{\mathrm{ni}} - P_i)$ where, as sketched in Fig. S2, P_{ni} denotes one of the neighbor sites of the i-th site. In all calculations we used $d = 0.5$ nm, which is approximately the size of the unit cell[15, 16], namely the smallest spatial scale over which the polarization can vary. The elementary polarization sites are arranged in an overall square simulation domain, as depicted in Fig. S3. The domain-wise Landau free energy anisotropy coefficients α_i, β_i and γ_i are determined by the intrinsic coercive field E_{IN} and remnant polarization P_{R}.
The domain-to-domain variations of these parameters can be introduced via a statistical distribution of E_{IN} and/or P_{R}. Such a distribution is obtained by choosing a mean value and a standard deviation for both the remnant polarization P_{R} and the coercive field E_{IN}. After the statistical assignment of P_{R} and E_{IN} to a given polarization site, the corresponding anisotropy coefficients of the same site can be calculated. More specifically, in our simulations we made us of a Gaussian dispersion of E_{IN} (whereby σ_{Ec} denotes denotes the ratio between the standard deviation and the mean value), whereas we used no dispersion for P_{R}.

For the simulations of poly-crystalline ferroelectric materials (e.g. doped HfO_2), we used square domains and we stopped the propagation of the polarization reversal at the domain boundaries by setting k to zero, as depicted in Fig. S3a. For epitaxial materials (e.g. PZT samples from[3]), instead, we used periodic boundary conditions at the boundary of the domain. With such boundary conditions, the replicas of the simulated area effectively emulate the switching propagation in larger domains. Figure S3b displays the simulated area (central square) and the replicas corresponding to periodic boundary conditions.

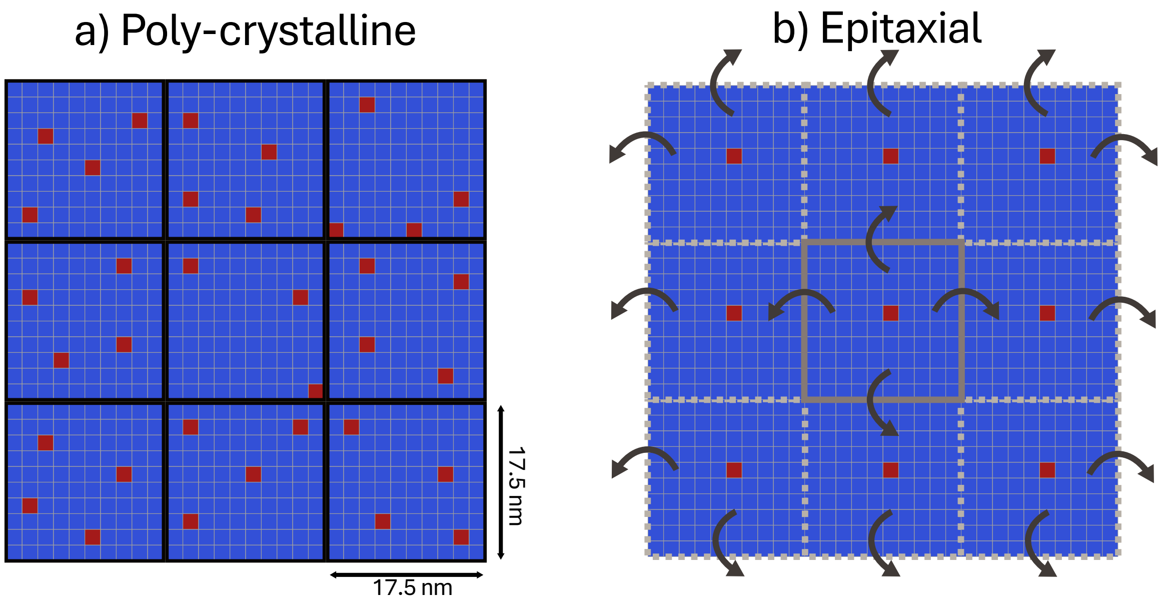

Figure S3. Sketch of the simulation arrangement for a poly-crystalline or an epitaxial material. Red squares indicate nucleated sites. (a) In poly-crystalline ferroelectrics, domains having a 17.5 nm side are separated by a dielectric border (solid black line). Switching propagation is blocked at such borders by setting $k = 0$. (b) Epitaxial materials have much larger grains, which are here emulated by using periodic boundary conditions.

All simulations in this work have been obtained with a 17.5 nm side domain (i.e. 35×35 elementary sites), unless otherwise mentioned.

3 Lorentzian distribution for Experimental Data in reference[17]

Figure S4 reports the Lorentzian distributions used for the simulations of the experimental datasets in[17]. As it can be seen, at higher E_{app} the half-width at half-maximum w is fairly independent of E_{app}, which resembles the behavior observed for epitaxial ferroelectrics and ascribed to a KAI-like behavior. At lower electric fields, however, w substantially increases and the resulting Lorentzian distributions display a profile spanning a very broad time range. This latter behaviour is usually associated to poly-crystalline ferroelectrics, and described in terms of the NLS model. These two different regions for the dependence of the half-width at half-maximum w of the applied field E_{app} are in agreement with the conclusions drawn by the authors of[17], who suggested that their samples exhibit two different reversal dynamics respectively at low and high E_{app}. Figure S4d further tests the **ExtNucl** LGD model against PR experiments for epitaxial $BiFeO_3$ thin films[17] showing again a good agreement between simulations and experimental data even for another type of ferroelectric material. All simulation parameters for this material are reported in Table S1

4 Simulation of arbitrary waveforms for the NLS scenario

The procedure to obtain the $P_T - E_{app}$ curve is similar to the one discussed in the Supplementary Information of[18], and it is based on the electric field–dependent Lorentzian distributions of the extrinsic nucleation times. More specifically, we extracted the parameters of the Lorentzian distributions of the nucleation times for different applied electric fields reported in[7]. Then, by proper parameters interpolation, we obtain Lorentzian distributions for the electric fields (see Fig. S5b) defined by the triangular voltage waveform (see Fig. S5a) used in our simulations. Such Lorentzian distributions are then used in our simulations to obtain the corresponding polarization reversal curves at a constant E_{app} magnitude, as illustrated in Fig. S5c, left y axis.

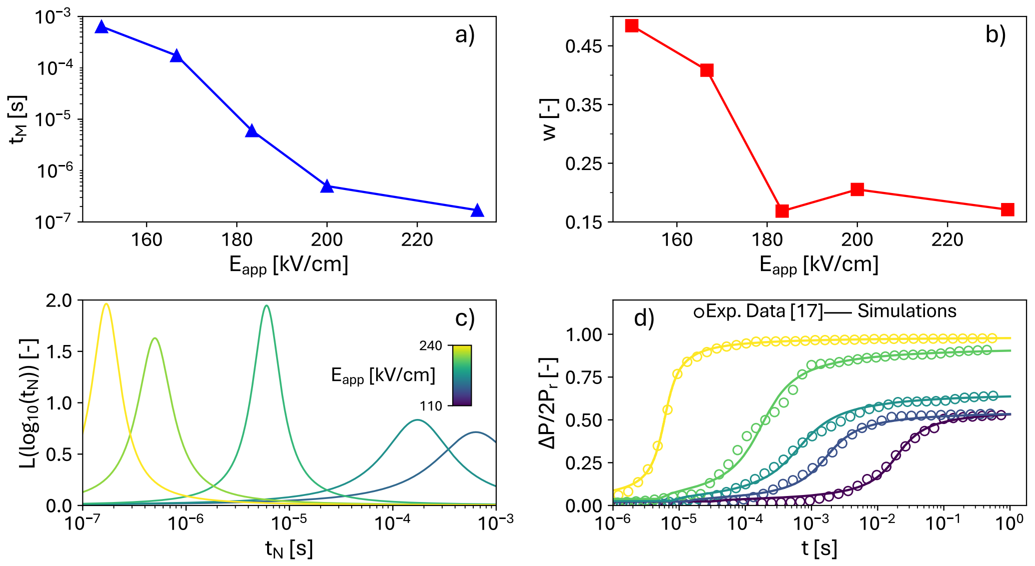

Figure S4. Lorentzian parameters extracted from experimental datasets in[17]. Mean nucleation time t_M (a), and half-width at half-maximum w (b) versus the applied field E_{app}. c) Lorentzian distributions corresponding to the t_M and w values in (a),(b), calculated by using Eq. (7) of the main text. d) Polarization reversal measurements and simulations for Epitaxial $BiFeO_3$ capacitors from.[17] All simulation parameters are reported in Table 1. In all **ExtNucl** simulations we used an extrinsic nucleation density of $3.26 \cdot 10^{11}$ cm^{-}2 and a nucleated area of 6.25 nm^2, which corresponds to a nucleated side of 2.5 nm.

The polarization reversal curves are then sampled at times t to obtain the time-dependent normalized switched polarization $P_{sig}/2P_R$ (Fig. S5c, right y axis). For example, the polarization reversal curve for $E_{app,1}$ is sampled at time t_1, the curve for $E_{app,2}$ is sampled at time t_2 and so on. The time-dependent normalized switched polarization is in turn used to extract the corresponding P_T versus E_{app} curve reported in Fig. S5d below and Fig. 4d in the main text. With this methodology we can obtain a quite good agreement between simulated and experimental P_T versus E_{app} curves even for materials whose polarization switching is governed by the rate of extrinsic nucleations.

	ExtNucl: LGD parameters. Data from[17] for epitaxial BFO. Simulations in Fig. S4d
E_{IN} [MV/cm]	0.25
σ_{EIN} [%]	1
P_R [μC/cm^2]	75
E_a [MV/cm]	$0.29@E_{app} < 0.18$ MV/cm<br>$0.74@E_{app} \geq 0.18$ MV/cm
ρ_0 [Ωm]	0.01
k [m^3/F]	$4.55 \cdot 10^{-}11$
α [m/F],<br>β [m^5/(FC^2)],<br>γ [m^9/(FC^4)]	$-9.39 \cdot 10^3$,<br>$-2.90 \cdot 10^7$,<br>$1.76 \cdot 10^7$

Table S1. Parameters for the LGD model extracted from a comparison between our simulations and several sets of experimental data. Parameters for the IntNucl LGD scenario correspond to comparisons in Fig. 2, whereas parameters for the ExtNucl LGD refer to Fig. 4. E_{IN} is the mean value of the coercive field for intrinsic nucleation and $\sigma_{E_{IN}}$ is the corresponding standard deviation (normalized to E_{IN}) for a site-to-site Gaussian distribution of the E_{IN} values. P_R and k are defined in the text, while ρ_0 and E_a describe the ρ dependence on E_{app} according to Merz' law in Eq. (5).

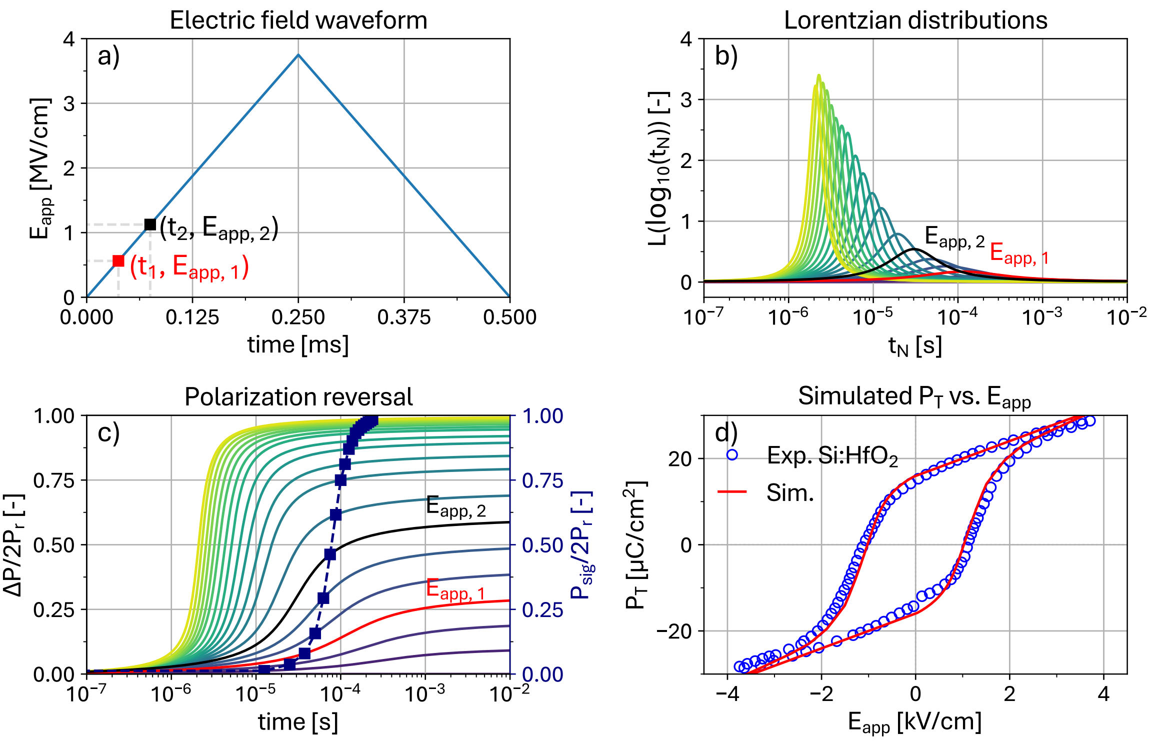

Figure S5. Simulation flow for arbitrary voltage waveforms. a) Arbitrary applied electric field E_{app} verus time; at each time step t, for example t_1 and t_2, we define the corresponding E_{app}: $E_{app,1}$ and $E_{app,2}$. b) Lorentzian distributions of nucleation times obtained for the arbitrary electric fields with parameters interpolated from experimental data. The Lorentzian distributions corresponding to $E_{app,1}$ and $E_{app,2}$ are highlighted in red and black. c) Simulated polarization reversal curves using the Lorentzian distributions in b), where the polarization reversal curves for $E_{app,1}$ and $E_{app,2}$ are again highlighted in red and black. d) corresponding P_T versus E_{app} characteristic for a triangular E_{app} waveform.

References

1. Ishibashi, Y. & Takagi, Y. Note on Ferroelectric Domain Switching. *J. Phys. Soc. Jpn.* **31**, 506–510, DOI: 10.1143/JPSJ.31.506 (1971). https://doi.org/10.1143/JPSJ.31.506.
2. Tagantsev, A. K., Stolichnov, I., Setter, N., Cross, J. S. & Tsukada, M. Non-Kolmogorov-Avrami switching kinetics in ferroelectric thin films. *Phys. Rev. B* **66**, 214109, DOI: 10.1103/PhysRevB.66.214109 (2002).
3. So, Y. W., Kim, D. J., Noh, T. W., Yoon, J.-G. & Song, T. K. Polarization switching kinetics of epitaxial $Pb(Zr_{0.4}Ti_{0.6})O_3$ thin films. *Appl. Phys. Lett.* **86**, 092905, DOI: 10.1063/1.1870126 (2005). https://pubs.aip.org/aip/apl/article-pdf/doi/10.1063/1.1870126/13224456/092905_1_online.pdf.
4. Buragohain, P. *et al.* Effect of Film Microstructure on Domain Nucleation and Intrinsic Switching in Ferroelectric Y:HfO_2 Thin Film Capacitors. *Adv. Funct. Mater.* **32**, 2108876, DOI: https://doi.org/10.1002/adfm.202108876 (2022). https://onlinelibrary.wiley.com/doi/pdf/10.1002/adfm.202108876.
5. Merz, W. J. Domain Formation and Domain Wall Motions in Ferroelectric $batio_3$ Single Crystals. *Phys. Rev.* **95**, 690–698, DOI: 10.1103/PhysRev.95.690 (1954).
6. Li, Z. *et al.* Grain size effect on piezoelectric properties of rhombohedral lead zirconate titanate ceramics. *Ceram. Int.* **49**, 27733–27741, DOI: https://doi.org/10.1016/j.ceramint.2023.05.209 (2023).
7. Lee, K. *et al.* Stable Subloop Behavior in Ferroelectric Si-Doped hfo2. *ACS Appl. Mater. & Interfaces* **11**, 38929–38936, DOI: 10.1021/acsami.9b12878 (2019). PMID: 31576734, https://doi.org/10.1021/acsami.9b12878.
8. Jo, J. Y. *et al.* Domain Switching Kinetics in Disordered Ferroelectric Thin Films. *Phys. Rev. Lett.* **99**, 267602, DOI: 10.1103/PhysRevLett.99.267602 (2007).
9. Zhou, S. *et al.* Van der Waals layered ferroelectric cuinp2s6: Physical properties and device applications. *Front. Phys.* **16**, 13301, DOI: 10.1007/s11467-020-0986-0 (2020).
10. Lee, D. H. *et al.* Effect of residual impurities on polarization switching kinetics in atomic-layer-deposited ferroelectric $Hf_{0.5}Zr_{0.5}O_2$ thin films. *Acta Materialia* **222**, 117405, DOI: https://doi.org/10.1016/j.actamat.2021.117405 (2022).
11. Rollo, T., Blanchini, F., Giordano, G., Specogna, R. & Esseni, D. Stabilization of negative capacitance in ferroelectric capacitors with and without a metal interlayer. *Nanoscale* **12,**, 6121–6129, DOI: 10.1039/c9nr09470a (2020).
12. Hoffmann, M. *et al.* Intrinsic Nature of Negative Capacitance in Multidomain $Hf_{0.5}Zr_{0.5}O_2$-Based Ferroelectric/Dielectric Heterostructures. *Adv. Funct. Mater.* **32**, 2108494, DOI: https://doi.org/10.1002/adfm.202108494 (2022). https://onlinelibrary.wiley.com/doi/pdf/10.1002/adfm.202108494.
13. Segatto, M., Fontanini, R., Driussi, F., Lizzit, D. & Esseni, D. Limitations to Electrical Probing of Spontaneous Polarization in Ferroelectric-Dielectric Heterostructures. *IEEE J. Electron Devices Soc.* **10**, 324–333, DOI: 10.1109/JEDS.2022.3164652 (2022).
14. Fontanini, R. *et al.* Interplay between charge trapping and polarization switching in beol-compatible bilayer ferroelectric tunnel junctions. *IEEE J. Electron Devices Soc.* **10**, 593–599, DOI: 10.1109/JEDS.2022.3171217 (2022).
15. Durruthy-Rodríguez, M. D., Gervacio-Arciniega, J. J., Hernández-García, M. & Yáñez-Limón, J. M. Photoluminescence characteristics of soft PZT 53/47 ceramic doped at A and/or B sites. *J. Adv. Ceram.* **7**, 109–116, DOI: 10.1007/s40145-018-0262-8 (2018).
16. Lee, J. *et al.* Role of oxygen vacancies in ferroelectric or resistive switching hafnium oxide. *Nano Convergence* **10**, 55, DOI: 10.1186/s40580-023-00403-4 (2023).

17. Pantel, D. *et al.* Switching kinetics in epitaxial BiFeO3 thin films. *J. Appl. Phys.* **107**, 084111, DOI: 10.1063/1.3392884 (2010). https://pubs.aip.org/aip/jap/article-pdf/doi/10.1063/1.3392884/13651214/084111_1_online.pdf.

18. Park, H. W. *et al.* Polarizing and depolarizing charge injection through a thin dielectric layer in a ferroelectric–dielectric bilayer. *Nanoscale* **13**, 2556–2572, DOI: 10.1039/D0NR07597C (2021).
